# Efficient structure learning of gene regulatory networks with Bayesian active learning

**DOI:** 10.1186/s12859-025-06149-6

**Published:** 2025-06-03

**Authors:** Dániel Sándor, Péter Antal

**Affiliations:** Department of Artificial Intelligence and Systems Engineering, Budapest University of Technology and Economics, Műegyetem rkp. 3, Budapest, 1111 Hungary

**Keywords:** Gene regulatory network, Structure learning, Active learning, Experiment design

## Abstract

**Background:**

Gene regulatory network modeling is a complex structure learning problem that involves both observational data analysis and experimental interventions. Bayesian causal discovery provides a principled framework for modeling observational data, generating posterior distributions that best represent the underlying structure. While recent algorithms offer efficient and accurate structure learning, integrating experiment design can further enhance predictive performance.

**Results:**

We introduce novel acquisition functions for experiment design in gene expression data, leveraging active learning in both Essential Graph and Graphical Model spaces. We evaluate scalable structure learning algorithms within an active learning framework to optimize intervention selection. Our study explores existing active learning strategies, adapts techniques from other domains to structure learning, and proposes a novel approach using Equivalence Class Entropy Sampling (ECES) and Equivalence Class BALD Sampling (EBALD). Using DREAM4’s Gene Net Weaver and Sachs protein signaling data, we assess the effectiveness of different strategies in improving network learning.

**Conclusion:**

Existing Bayesian experiment design strategies often overlook the Essential Graph structure, making inference more challenging due to the large number of possible graphs. Our results demonstrate that integrating active learning into structure learning algorithms can significantly improve performance, offering a scalable and effective approach for gene regulatory network discovery.

## Background

Gene Regulatory Networks (GRNs) are complex computational representations of biological interactions that govern cellular processes. Precise computational modeling of these networks enables targeted interventions, potentially addressing pathological mechanisms in diseases, cellular aging, and developmental disorders. Efficient intervention enables the regulation of biological processes, potentially correcting pathological mechanisms (e.g., curing diseases or mitigating aging). The structure of the network consists of gene nodes that form a directed graph. The directed edges represent regulatory relationships between genes. To identify these relationships, we can look at gene expression (GEX) levels.

GEX refers to genes exerting their effects on the phenotype and contributing to their biological functions. This is done through RNA sequences, which regulate other genes and the biological processes connected to the given phenotype. The level of GEX is quantified by counts of RNA sequences in a given timeframe. These expression levels can be used to infer regulatory relationships between genes.

One suitable method to learn GRN structure from GEX data is causal discovery [1–3]. In this context, we view the GRN as a Directed Acyclic Graph (DAG), with genes as nodes and directed regulatory relationships as edges. We try to learn the structure of a Probabilistic Graphical Model (PGM) from the GEX data. For this task, we chose two algorithms: BayesDAG [4] and Generative Flow Networks (GFN) with a DAG learning focus [5]. The reason we used these algorithms is that they are scalable and efficient with large datasets, and they are both able to sample models from the posterior distribution (not just point estimates), thus allowing for Bayesian Active Learning.

The efficient learning of high-dimensional data is important as the initial training of the models can include several hundred or even thousands of data samples, which is usually not feasible with traditional score-based or constraint-based methods. This new class of models is efficient and performs reasonably well under ideal conditions. However, they can be subject to known biases, which might deteriorate their performance. Active learning is, therefore, critical, as it enables the generation of new samples to improve model performance.

Bayesian learning is a valuable approach for optimal experiment design or active learning since it allows us to understand the distribution of the models. By quantifying the uncertainty of the DAG distribution, we can select the edges that are the most uncertain in a classical setting. Creating samples by gene knockouts can distinguish given edge directions and allow the model to learn faster or improve its performance. BayesDAG uses gradient-based Markov Chain Monte Carlo to sample DAGs from the posterior distribution, allowing us to use these for active learning. In the GFN, the DAG is generated sequentially, and by using flows, we can sample the distribution of DAGs to find out which edge has high uncertainty.

When these algorithms are run in a non-active setting, we can infer a distribution from them and sample the GRNs. Using active learning allows us to include experiments to enhance their performances with interventional data. The use of experiments under real-world conditions is expensive and time-consuming. Thus, we can only allow a few optimal experiments. These limited number of experiments must be selected carefully in a way that can improve the model the most. This process corresponds to optimal experimental design, operationalized via active learning.

In active learning, the models are trained until convergence and then sampled to find places in the distribution where new data samples would be most usable. The selection of these samples is based on acquisition functions. These are criteria that can select possible inputs or experiments for an oracle to conduct based on the models’ current distribution.

Traditionally, for structure learning, Edge Entropy or similar functions are used. However, in our setting, we are interested in the concrete graph identified by interventional data. We thus need not only the Markov Equivalence class (represented by the Essential Graph) but also the concrete instance of the DAG. To find this, we introduce Equivalence Class Entropy Sampling (*ECES*) and Equivalence Class-based Bayesian Active Learning By Disagreement (*EBALD*), which are modifications of existing acquisition functions to work in an equivalence class-based DAG learning setting.

### Related work

Bayesian network (BN) inference and structure learning are computationally intractable in both exact and stochastic settings [6–8]. Similarly, the sample complexity of learning from complete observations is prohibitively high, requiring an impractically large number of samples to reliably identify the observationally equivalent class [9, 10]. Learning in causal models presents even more significant challenges, encompassing both causal discovery (of structure) and causal inference (of effect strength), especially when dealing with incomplete data-frequently missing in a non-random fashion.

To address these difficulties, sequential learning methods, such as active learning, multi-armed bandits (MAB), and reinforcement learning (RL), have been successfully developed to improve both computational and sample efficiency [11]. These methods have also been applied in critical areas such as hyperparameter selection [12] and adaptive Monte Carlo simulations [13], both of which are pivotal for Bayesian inference and BN structure identification. Similarly, data point selection in case of highly incomplete observations remains a key consideration in multitask learning [14–16]. Furthermore, intervention selection (a.k.a. experimental design) represents an additional layer of automation, enabling efficient causal discovery and inference, even advancing towards the automation of scientific discovery [17–22]. Specifically, Lu et al. [23] applied multi-armed bandit algorithms explicitly to Bayesian network learning. For comprehensive overviews of reinforcement learning for Bayesian network learning and causality research, see the surveys by Deng et al. [24] and Zeng et al. [25].

Tong and Koller [26, 27] pioneered active learning in both parameter estimation and structure learning of Bayesian networks. Yoo and Cooper [28] proposed a comprehensive Bayesian decision-theoretic discovery framework for causal models (e.g., gene regulatory networks), optimizing intervention selection through experimental design. He and Geng [29], Hauser and Bühlmann [30] developed two-step approaches: first identifying observationally equivalent classes and then refining them into causal models (for recent improvements, see also [31–33]).

Rubenstein et al. [34] advanced local causal submodel learning using probabilistic active learning strategies. The Bayesian Active Learning by Disagreement (BALD) approach, proposed by Houlsby et al. [35], was later adapted for causal inference by Jesson et al. [36]. In this paper, we extend this method-along with the approaches of Kirsch et al. [37] and Gal et al. [38]-to the context of Bayesian network structure learning.

The availability of gene expression (GEX) data in the early 2000 s initiated the inference of gene regulatory networks using Bayesian Networks (BNs) [39] and Dynamic Bayesian Networks [40]. The advent of genetic perturbation data, as a natural repertoire of genetic interventions, enabled more profound insights into the genetics of GEX and its mediator role toward complex phenotypes [41]. Regulatory information about transcription factor binding sites (TFBSs), microRNAs, and other regulatory elements facilitated the integration of complex regulatory information into tissue-specific regulatory discovery [42].

The emergence of single-cell RNA sequencing (scRNA-seq) combined with chromatin accessibility profiling revolutionized the field by enabling the observation of regulatory mechanisms at an individual cell level, capturing both regulatory factors and their precise manifestation [43–49]. More recently, the development of inferring RNA velocity in sequencing has made it possible to study not only static regulatory snapshots but also the dynamics of gene regulation over time [50].

### Contributions

The main contributions in the paper are the following:

1. We adapt state-of-the-art active learning strategies to scalable structure learning and compare their performance to existing acquisition functions.
2. We introduce *ECES* and *EBALD* for structure learning in Equivalence Classes.
3. We evaluate modern, scalable Bayesian approaches for learning structure in GRNs. And measure their performance.

## Methods

**Fig. 1:**
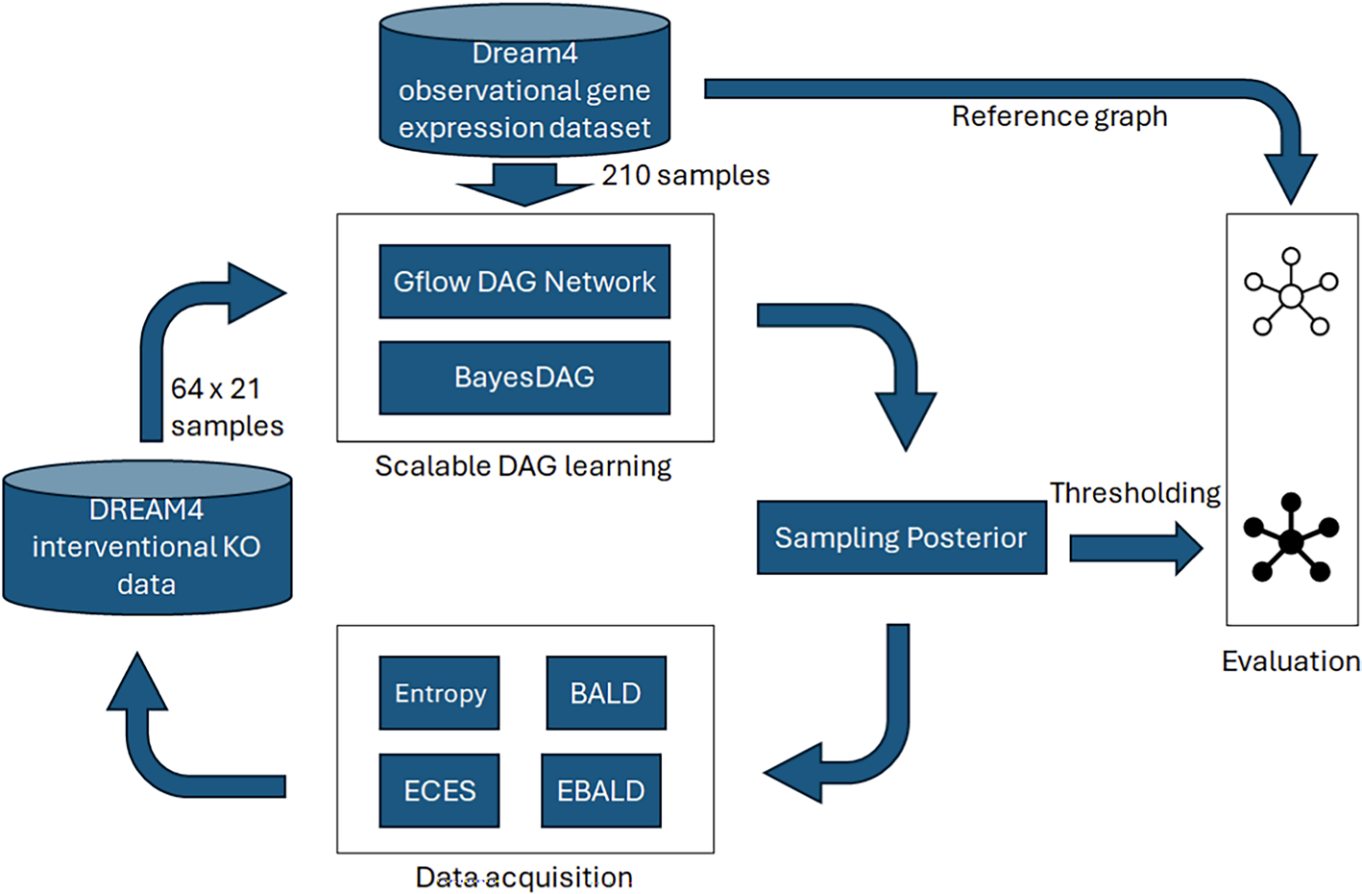
Active learning in scalable learning of GRNs

When constructing the GRN structure, we use observational data from the DREAM4 challenge’s example model from Gene Net Weaver (GNW) [51]. In each setup, pretraining rounds are performed by the structure learning algorithms. After the models have converged, they are evaluated by the acquisition functions to get a clear picture of the learned posterior and its uncertainty. When the acquisition functions choose the knockout experiments to simulate, they are retrieved from the previously simulated GNW model and added to the training data. Then, the models are retrained, and the new round begins until the desired accuracy is reached or the simulated resource for the experiments runs out. This process is summarized in Fig. 1.

### Scalable Bayesian structure learning for GRN

An advanced computational approach to causal discovery is utilizing continuous optimization techniques [4, 52, 53]. The challenge here is the formulation of the DAG constraint; by using continuous optimization, these methods circumvent the difficulties caused by the exponentially large discrete state space. Bayesian causal discovery is useful if we are interested in the distribution of potential models, which is needed in the case of active experiment design [38].

#### BayesDAG

BayesDAG was the first scalable algorithm available for Bayesian learning and sampling directly from the posterior without applying DAG constraints after sampling. It uses stochastic gradient Markov Chain Monte Carlo (SG-MCMC) and Variational Inference (VI) to generate a posterior distribution. While the learning algorithm heavily relies on the DAG No-Curl [53] method.

To our knowledge, BayesDAG has not yet been applied to GRN structure learning; thus, choosing hyperparameters is challenging. As in most cases, structure learning algorithms work on normalized data, which aligns with the DREAM4 dataset. The advantage of BayesDAG to previous methods is enhanced computational scalability with probabilistic uncertainty quantification, enabling the analysis of high-dimensional networks with low complexity. In theory, the method also allows for introducing edge priors, which in the case of GRNs can offer a significant advantage over other forms of scalable structure learning. However, we only consider Uniform edge priors to maintain a fair comparison.

#### Generative flow networks

Generative Flow Networks (GFN) [54] are probabilistic generative networks for discrete structure inference using energy-based optimization techniques. GFN generates a distribution based on the energy function; this distribution can be viewed similarly as the posterior of the BayesDAG model. In this paper, we use the DAG-GFlowNet implementation [5], which is explicitly designed to support DAG sampling from the network. In this method, the DAG constraint is nonexistent as the structure is generated one edge at a time, guaranteeing its DAG structure. The posterior is sampled from these generated DAGs based on the flow functions. GFN have been used for generation of GRNs [55]; however, this Bayesian approach supports sampling from the posterior of edges, which is a requirement for active learning, and it generates DAGs, thus it stays comparable to BayesDAG.

#### Sampling the models

In each case, the models give us the posterior distribution of DAGs. For the active learning step, we aim to computationally characterize directional dependencies in the gene regulatory network. To this end, we sample the model to approximate the posterior distribution for the edges. We use these sampled edges to conduct active learning and the resulting DAGs based on the edge samples to compare the methods.

#### Interventional knockout data

Our work uses experiments to specify a DAG as best as possible from interventional data. This is possible because the experiments accomplish a controlled intervention in the variables, which is used to specify variable relationships and edge directions.

In these experiments, we use GeneNetWeaver to simulate multiple single-gene knockout interventions. These are done by simulating the knockout and perturbing the graph with outside signals. After this, we observe the expression levels in a time series. The simulated knockout will be sampled from this time series to simulate the experiment’s randomness. We append the simulated experiments to the observational data to enrich the training set size. After training, the edge directions of the Essential Graph can be determined based on the simulations conducted.

### Active learning

We use active learning to specify the DAG structure and complement the learning process. The theoretical benefit of this lies in two key areas:

- Using interventional data to identify the DAG structure fully [56].
- Making the learning process more efficient by having the optimal samples available to specify edges, resulting in faster convergence [27].

The use of interventional experiments is done the following way: After training the learning algorithms until convergence, we specify the posterior distribution over the edges *P*(*W*|*data*), with *W* representing the adjacency matrix. Historically, the entropy over edges is used to select the target for intervention [27]. Edge Entropy is given by considering the entropy over the three states of the distribution: edge in direction $X_i \rightarrow X_j$, edge in direction $X_i \leftarrow X_j$ or no edge between $X_i$ and $X_j$.

1 $$\begin{aligned} \begin{aligned} H(X_i \leftrightarrow X_j)=-P(X_i \rightarrow X_j) log P(X_i \rightarrow X_j) \\ -P(X_i \leftarrow X_j) log P(X_i \leftarrow X_j) \\ -P(X_i \quad X_j) log P(X_i \quad X_j) \end{aligned} \end{aligned}$$

In practice, we can sample the posterior *W*, thus we calculate the entropy of the expected edges, given by:

2 $$\begin{aligned} \begin{aligned} H({\mathbb {E}}[X_i \leftrightarrow X_j])=-P({\mathbb {E}}[W_{ij} = 1] ) log P({\mathbb {E}}[W_{ij} = 1 ]) \\ -P({\mathbb {E}}[W_{ji} = 1]) log P({\mathbb {E}}[W_{ji} = 1]) \\ -P({\mathbb {E}}[W_{ij} = 0], {\mathbb {E}}[W_{ji} = 0]) log P({\mathbb {E}}[W_{ij} = 0], {\mathbb {E}}[W_{ji} = 0]) \end{aligned} \end{aligned}$$

We assume that the resulting graph is a DAG; thus, we omit the ${\mathbb {E}}[W_{ji}] = 0$ and ${\mathbb {E}}[W_{ij}] = 0$ parts from the first two parts of Eq. 2 and for the same reason we omit the fourth state, where the edge would be present in both directions.

If we want to have an acquisition function applicable to causal structures to select from single gene knockout experiments, we need to sum over $H(X_i \leftrightarrow X_j)$:

3 $$\begin{aligned} \sum _j H({\mathbb {E}}[X_i \leftrightarrow X_j]) \end{aligned}$$

Summing over the index *j* means we get the nodes with the most “problematic” edges incoming, meaning those with the largest sum of incoming Edge Entropies. We want to intervene (knock out) at the parents of these nodes. To find parents that are good candidates, we have to consider two factors: the connecting edge is certain, meaning ($H({\mathbb {E}}[X_i \leftrightarrow X_j])$ is low. And they exist with a high probability, meaning $P({\mathbb {E}}[W_{ij}] = 1 )$ is high.

The final form of the acquisition function we can use is:

4 $$\begin{aligned} \text {arg}\,\max [\sum _i [(1-H({\mathbb {E}}[X_i \leftrightarrow X_j])) \circ P({\mathbb {E}}[W_{ij} = 1] ) \circ \sum _j H({\mathbb {E}}[X_i \leftrightarrow X_j])]] \end{aligned}$$

**Fig. 2:**
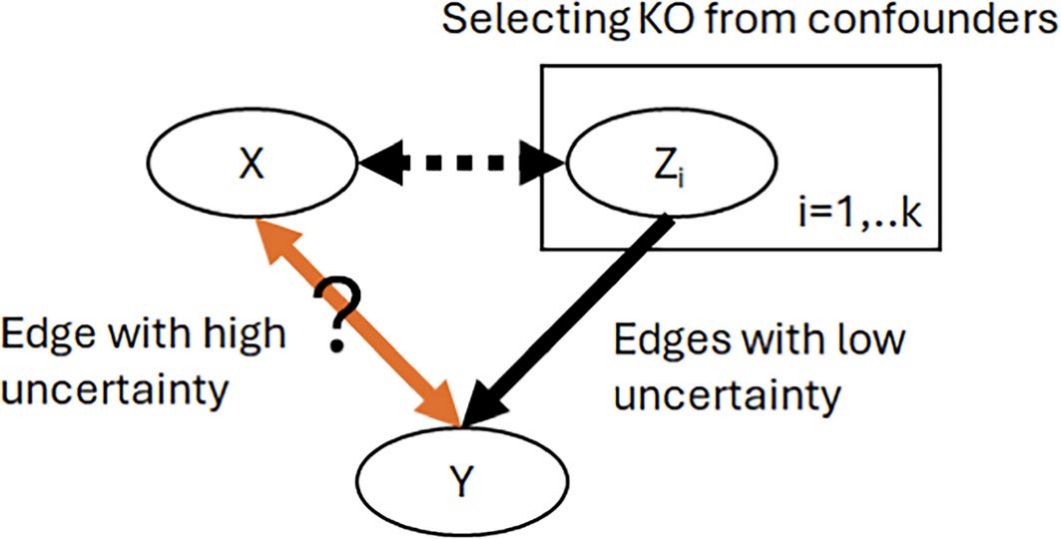
The acquisition function needs to find good candidates for Knockout Experiments. This means that we identify edges with high uncertainty (or entropy, $\sum _j H({\mathbb {E}}[X_i \leftrightarrow X_j])]$) and select from the confounder variables (ones with edges of high certainty and high probability $\sum _i [(1-H({\mathbb {E}}[X_i \leftrightarrow X_j])) \circ P({\mathbb {E}}[W_{ij} = 1] )).$

This can find the nodes with the most uncertain dependencies of its parents, as shown in Fig. 2. To find good candidates for knockouts, we can select from the parents of the found nodes. Choosing based on Edge Entropy is straightforward, and we can see that it results in new samples that can reduce the overall entropy. However, in several other domains, more refined acquisition functions have been used [37, 38] with notable success.

#### Structure BALD

Bayesian Active Learning by Disagreement (BALD) is a method that builds on entropy, but it takes into account the disagreement between the averaged model. In structure learning, this means that if an edge is predicted to have a high probability in one sample and low probability in another, their disagreement causes the function to select this edge with higher probability. We apply *BALD* for the space of directed edges in a DAG, similarly as we use edge entropy:

5 $$\begin{aligned} \begin{aligned} BALD(X_i \leftrightarrow X_j)= H({\mathbb {E}}[X_i \leftrightarrow X_j]) - {\mathbb {E}}[H(X_i \leftrightarrow X_j)] \end{aligned} \end{aligned}$$

*BALD* decomposes the mutual information between the model and the data using the entropy and the expected entropy over the sampled models. In practice, we can do this by calculating the same entropy in the same way as Edge Entropy and subtracting the expected value of the individual sampled model’s edge entropies. Where the first term is calculated according to Eq. 2, and the second term is given by averaging entropy over sampled models:

6 $$\begin{aligned} \begin{aligned} {\mathbb {E}}[H(X_i \leftrightarrow X_j)] = {\mathbb {E}}[-P( W_{ij} = 1 ) log P(W_{ij} = 1 ) \\ -P(W_{ji} = 1) log P(W_{ji} = 1) \\ -P(W_{ij} = 0, W_{ji} = 0) log P(W_{ij} = 0, W_{ji} = 0)] \end{aligned} \end{aligned}$$

This way, we expect the results to be more stable, as experienced previously in other domains.

#### Active learning in the essential graph space

Without prior knowledge, observational data can identify the structure of a gene regulatory network only up to its Markov equivalence class, leaving edge directions ambiguous. In contrast, interventional experiments enable the exact identification of the underlying DAG structure by resolving these directional uncertainties. This distinction is particularly important in our case, as edge directions determine the causal interactions within the regulatory network. To find out the directions in the Essential Graph, we need interventions that target nodes with undirected edges; thus, we introduce active learning in the space of Essential Graphs.

In most cases, a fully specified network is a desirable learning outcome. Thus, if we implement the previously defined active learning procedures but for the partially directed (PDAG) Essential Graph, we can consider the presence and direction of edges as distinct problems and expect more specified graphs as a result.

To do this, we first show Equivalence Class Entropy Sampling (ECES) based on Edge Entropy. If we consider a PDAG versus a DAG, the distribution of an edge can have four states: Edge in direction $X_i \rightarrow X_j$, edge in direction $X_i \leftarrow X_j$, no edge between $X_i$ and $X_j$ and undirected edge $X_i - X_j$, which was not present in DAGs previously. We can calculate the entropy of this distribution similarly as previously:

7 $$\begin{aligned} \begin{aligned} H(X_i \leftrightarrow X_j)=-P(X_i \rightarrow X_j) log P(X_i \rightarrow X_j) \\ -P(X_i \leftarrow X_j) log P(X_i \leftarrow X_j) \\ -P(X_i \quad X_j) log P(X_i \quad X_j) \\ -P(X_i - X_j) log P(X_i - X_j) \end{aligned} \end{aligned}$$

However, if the $P(X_i - X_j)$ probability is high, then we would want to know the direction, thus the previously described metric is not necessarily a good acquisition function. To improve this, we sample the entropies based on the $P(X_i - X_j)$ probability, and in the cases where the probability of an undirected edge is high, we only consider the entropy over the distribution of the edges’ direction:

8 $$\begin{aligned} \begin{aligned} H(X_i \leftarrow \rightarrow X_j)=-P(X_i \rightarrow X_j) log P(X_i \rightarrow X_j) \\ -P(X_i \leftarrow X_j) log P(X_i \leftarrow X_j) \end{aligned} \end{aligned}$$

For the acquisition step, we sample *U*, where $U_{ij} \sim P(X_i - X_j)$.

The ECES score is then computed as:

9 $$\begin{aligned} ECES_{ij} = H(X_i \leftarrow \rightarrow X_j) U_{ij} + H({\mathbb {E}}[X_i \leftrightarrow X_j])\lnot U_{ij} \end{aligned}$$

Here $\lnot U_{ij}$ is the element-wise complement (logical NOT) of the *U* matrix, thus selecting the complementary elements in the second sum. The choice of the final acquisition function form is based on Eq. 4, we multiply high probabilities with low uncertainties to find suitable parents (for knockouts), and we multiply this with the neighbors, which have edges with high uncertainty quantified by ECES scores:

10 $$\begin{aligned} \text {arg}\,\max [\sum _i [(1-ECES_{ij}) \circ P({\mathbb {E}}[W_{ij} = 1 ]) \circ \sum _j ECES_{ij}]] \end{aligned}$$

Similarly, for EBALD we can calculate the BALD metric over the space of Essential Graphs:

11 $$\begin{aligned} \begin{aligned} EBALD_{ij} = H({\mathbb {E}}[X_i \leftarrow \rightarrow X_j]) - {\mathbb {E}}[H(X_i \leftarrow \rightarrow X_j)] U_{ij} + \\ H({\mathbb {E}}[X_i \leftrightarrow X_j]) - {\mathbb {E}}[H(X_i \leftrightarrow X_j)]\lnot U_{ij} \end{aligned} \end{aligned}$$

Thus, the EBALD acquisition function is the following:

12 $$\begin{aligned} \text {arg}\,\max [\sum _i [(1-EBALD_{ij}) \circ P({\mathbb {E}}[W_{ij} = 1] ) \circ \sum _j EBALD_{ij}]] \end{aligned}$$

## Results

We conduct experiments to test the efficiency of each acquisition function and structure estimator. We evaluate the number of generated edges (edges, that are non-zero, NNZ), the expected structural hamming distance (SHD) between the DAGs (both in a directed and undirected case, to see how well causality is represented), and the expected SHD between the PDAGs, to see how well we can identify the equivalence classes.

### Description of data

We conduct our experiments on simulated and real data. We use simulated data to test the results of knockout interventions on gene regulatory networks. For real-world validation, we use the Sachs protein signaling dataset to compare our methods against state-of-the-art approaches. While this dataset does not represent a gene regulatory network (GRN) per se, the underlying protein signaling network exhibits structural properties similar to GRNs and is commonly used to benchmark structure learning algorithms. Finally, we present our results based on a Caenorhabditis Elegans aging atlas [57]. In this case, no interventional data is available; instead, we demonstrate how our proposed acquisition strategies could guide future interventions to further resolve the network structure. We compare this with existing literature to validate our findings.

#### Simulated data

**Fig. 3:**
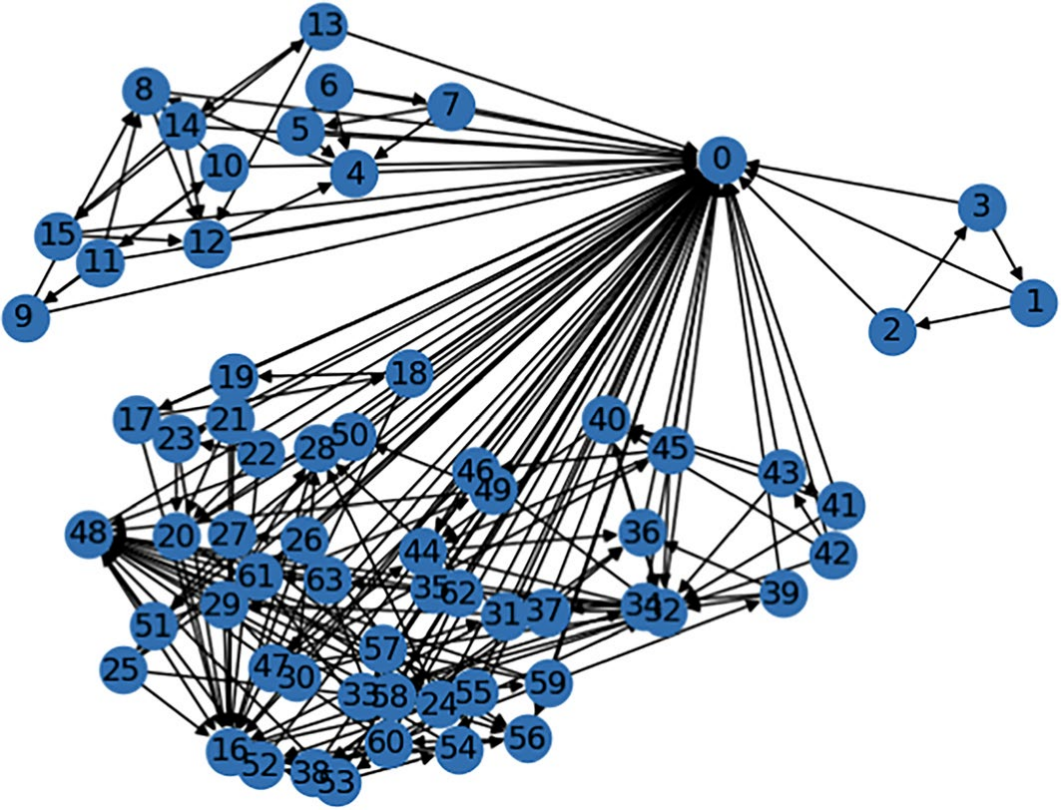
Ground truth graph from which the data is generated [51]

The simulated experiments are conducted on the example network of Gene Net Weaver, which contains 64 nodes and 207 edges. The structure of the network can be seen in Fig. 3. This means it is a sparse network, which introduces computational challenges in distinguishing signal from stochastic noise in representations. In the observational setting, the models have access to the observational time series data, which results from introducing perturbation to the network and waiting for it to stabilize. The observational data contains 10 such time series, each measured at 21 distinct time points, to get a total of 210 samples for the GEX levels of the 64 genes. The sizes of the data sets are summarized in Table 1. In the interventional setting, the data generation process is identical, except that specific genes are inactivated. In this setting, the available data for one gene is one time series (consisting of 21 samples). Thus, the number of interventional samples is 1,344 (64x21). The reference PDAGs are generated by removing 21 edges from the original reference graph (as this is not a DAG), then it is converted to a PDAG based on the Meek’s rules [58].

#### Real data

In the case of real data, we use the Sachs dataset [59], which is based on protein-protein interactions. It serves as a good baseline for Gene Regulations as the data consists of expression levels, and the Goal is to find the DAG signaling network. The dataset was discretized to 3 levels: low, medium, and high expressions. We used the interventional data as the possible experiments; however, in the case of this setup, interventions deviate from standard gene knockout experimental protocols. The sizes of the datasets are seen in Table 1. In this case, 10 interventions were chosen in every iteration of the algorithm randomly from the pool of samples with the given intervention.

For the Aging Atlas [57], we used a curated number of genes, mainly those mentioned to influence age directly. From this, we trained a Generative Flow-based model and checked for each acquisition function, which genes are worth exploring by knockout experiments.

**Table 1:** Sizes of the two datasets

	Variables (D)	Possible interventions	Total samples
GNW Observational data	64	NA	210
GNW Interventional data	64	64	1344
Sachs observational	11	NA	853
Sachs interventional	11	6	5400

The GNW observational data set contains 10 time series with different perturbations measured at 21 time points. The GNW interventional data set contains one time series for each of the possible 64 genes. And every gene’s expression is measured for every sample. The Sachs dataset contains 11 proteins, with the interventions measured for 6 of them.

### Results on observational data

In the observational case, BayesDAG was trained for 64 epochs starting from a sparsely initialized graph with large lambda (to result in extremely sparse graphs). GFlow net is trained for 10,000 epochs with a pretraining of 1000 epochs. Both models converge and find an optimum, but as we can see in Table 2 their results are different.

**Table 2:** Results on observational data

Data	Model	SHD (D)	SHD (U)	PDAG SHD	NNZ
GNW	BayesDAG	329.69	320.97	311.11	144.06
	GFlow net	320.97	314.88	302.27	134.97
Sachs	BayesDAG	19.22	18.53	19.79	4.21
	GFlow net	17.66	17.39	17.8	1.45
	BCD Nets	14.7	NA	14.0	9.2

Similar performance from both algorithms after initial training in the observational setting. Based on the metrics: Directed Structural Hamming Distance (SHD (D)), Undirected Structural Hamming Distance (SHD (U)), Structural Hamming Distance for the Essential Graphs (PDAG SHD), Non-zero edges predicted (NNZ).

As seen in Table 2, both the BayesDAG and the GFlow algorithms find a smaller than ideal number of edges, and most of the found edges are also incorrect, leading to low recall values in both cases. We compare our results with those of BCD-Nets, which are scaleable and currently present the best results in the scaleable algorithms. The methods as expected are close to the BCD-Nets performance but do not reach it, thus we know there is room for improvement, which can be utilized by the active strategies.

### Results with active learning in DAG space

Both algorithms are tested in the DAG space to see if improvement can be reached by introducing new experiments to the dataset and performing simulated knockouts. The baseline is a uniform selector that chooses each gene with an equal probability of knockout. We compare Edge Entropy and BALD in terms of identifying the structure with the previously discussed metrics.

**Table 3:** Results on interventional data with active learning in DAG space

Data	Model	SHD (D)	SHD (U)	PDAG SHD	NNZ
GNW	BayesDAG uniform	312.72	304.64	293.7	123.11
	BayesDAG entropy	327.89	322.5	309.19	144.16
	BayesDAG BALD	319.77	312.83	301.69	134.86
	GFlow net uniform	541.45	521.23	527.11	396.58
	GFlow net entropy	214.66	214.31	193.81	8.66
	GFlow net BALD	243.39	241.394	223.08	42.73
Sachs	BayesDAG uniform	19.16	18.69	19.41	4.0
	BayesDAG entropy	19.32	18.62	19.89	4.32
	BayesDAG BALD	19.71	19.03	20.29	4.76
	GFlow net uniform	48.03	38.0	53.59	55.0
	GFlow net entropy	17.09	17.09	17.09	0.125
	GFlow net BALD	17.08	17.05	17.11	0.14

BayesDAG improves little with the addition of new data, the GFlow algorithm shows different behaviors under different acquisitions strategies: the uniform acquisition makes it overfit, entropy penalizes edges too much, but BALD can keep a balance between the number of edges and the correctness of edges. Based on the metrics: Directed Structural Hamming Distance (SHD (D)), Undirected Structural Hamming Distance (SHD (U)), Structural Hamming Distance for the Essential Graphs (PDAG SHD), Non-zero edges predicted (NNZ). In the case of the Sachs data, the main difference is between the models used for training. However, in the case of GFlow, only targeted interventions seem to give meaningful results.

#### BayesDAG and acquisition functions

**Fig. 4:**
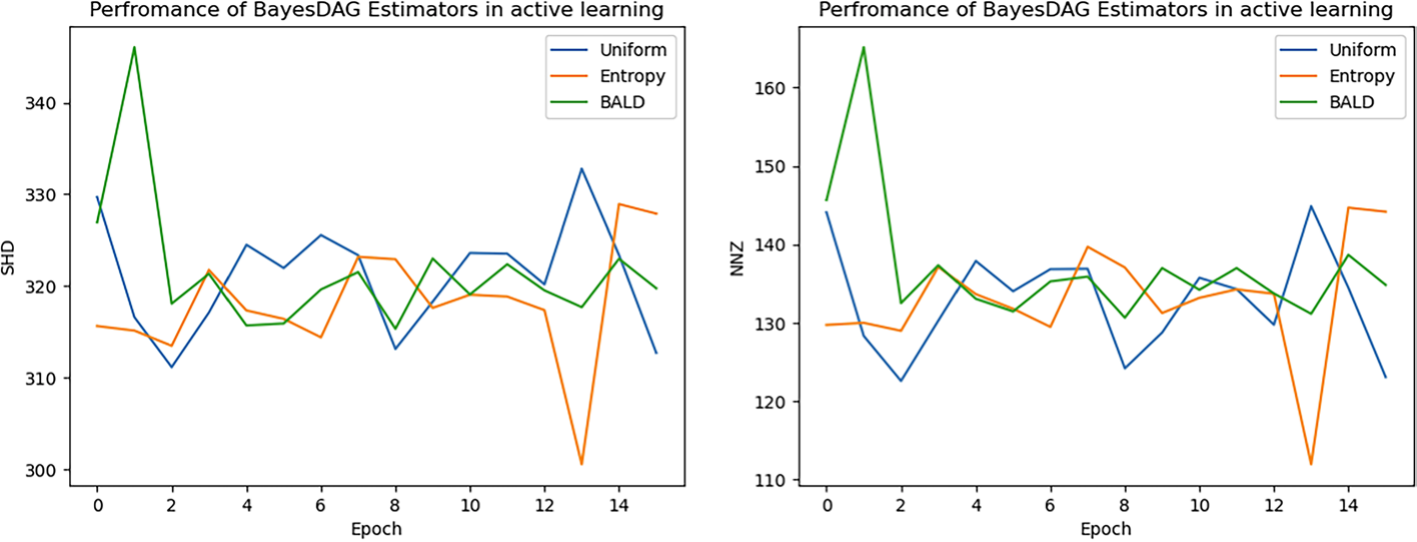
The Directed Structural Hamming Distance (D SHD) and the Non-zero edges (NNZ) metrics did not change significantly with the addition of new data in the BayesDAG algorithm, for the case of the GNW data

As Table 3 shows BayesDAG does not improve with the addition of interventional experiments, we see the same behavior in Fig. 4.

Adding new data does not impact the generated posterior distribution, leading to all sampled graphs having similar numbers and directions of edges. This means that the posterior found in the early stages of the learning process can persist.

#### GFlow Net and Acquisition Functions

**Fig. 5:**
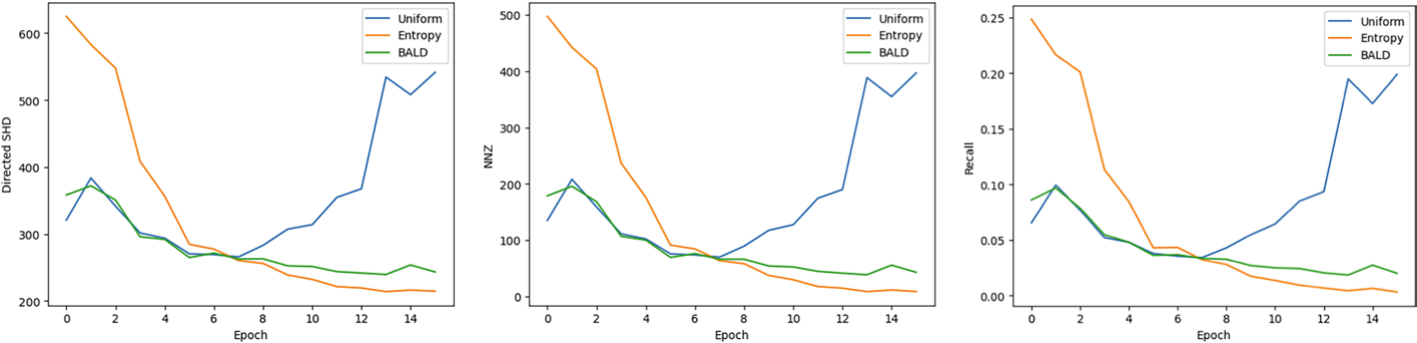
Different GFlow setups exhibit different behaviors in terms of Directed Structural Hamming Distance (D SHD) (left), the Non-zero edges (NNZ) (middle) and recall (right), uniform acquisition generates high number of edges to find more positives, Entropy converges to zero edges, BALD finds balance between edge numbers and accuracy

**Fig. 6:**
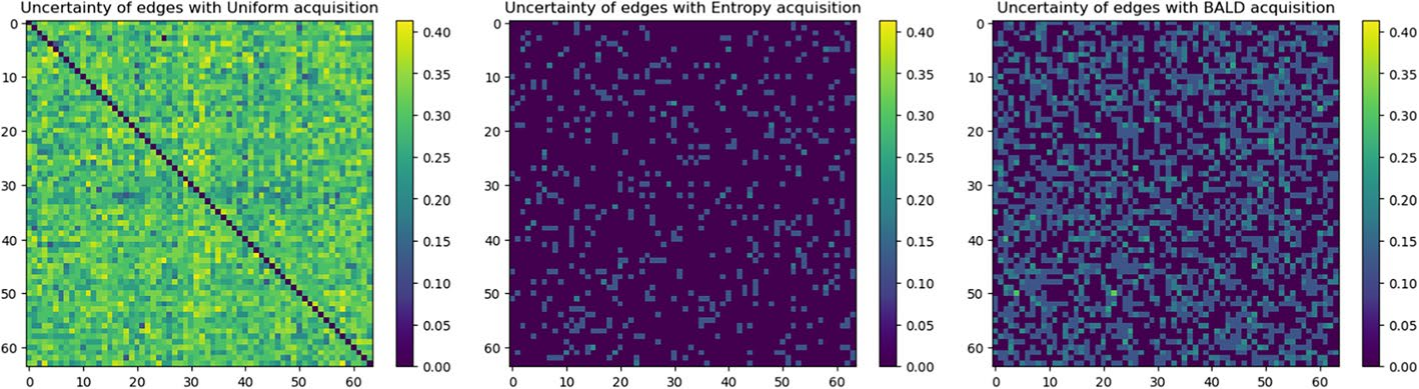
Uncertainty of edge distributions in different final models of GNW: uniform acquisition function (left) did not reduce uncertainty in predictions, Entropy (middle) had the most certain edges, BALD reduced completely in some blocks, and reduced it overall compared to the uniform approach

When looking at the Generative Flow Networks, we find that different acquisition functions lead to different results. In Fig. 5 we can see that directed SHD, NNZ and recall all correlate strongly in each model, thus we will discuss them jointly. With the addition of interventions, the uniform acquisition case starts to generate more edges. This might be because the uniform acquisition method had the most diverse pool selected by the end of the learning process. Thus, the edges in the final graph are still uncertain to a higher degree, which is confirmed in Fig. 6.

BALD and Edge Entropy both reduced uncertainty, which was their primary goal. Thus, both posteriors are more focused on their predictions. Entropy is the most certain, as most edges are predicted as non-existent. This might be explained by the fact that the acquisition relied so heavily on reducing uncertainty that confirming some initial low probability edges as non-existent made it converge to an optimum with no predicted edges. On the other hand, BALD relies on disagreement of predictors, which can only happen if there exist predictors with edges predicted (with significant probabilities); thus, it considered these more and found some samples that confirmed the existence of a few edges. This is only valid to an extent, as the number of edges is still minimal (see Fig. 5). To summarize, the uniform function does not reduce uncertainty, leading to too many predicted edges; Entropy overly reduces uncertainty, and BALD creates a balance between these while being closer to the Entropy-based result.

### Results with active learning in PDAG space

We compare ECES and EBALD in the PDAG space. We focus on whether they can correct ambiguous edge directions. The results are summarized in Table 4. In the case of the BayesDAG, the results did not improve significantly, although some changes are noticeable in the PDAG SHD scores.

**Table 4:** Results on interventional data with active learning in PDAG space

Data	Model	SHD (D)	SHD (U)	PDAG SHD	NNZ
GNW	BayesDAG ECES	324.06	318.06	305.08	138.0
	BayesDAG EBALD	327.75	318.64	308.86	141.61
	GFlow net ECES	231.78	230.52	211.11	28.58
	GFlow net EBALD	212.69	212.25	191.83	7.09
Sachs	BayesDAG ECES	19.22	18.58	19.58	3.94
	BayesDAG EBALD	19.19	18.28	19.79	4.56
	GFlow net ECES	17.11	17.09	17.10	0.125
	GFlow net EBALD	45.57	38.0	53.48	55.0

BayesDAG remains unaffected by the data acquisitions, the performance of the GFlow algorithmsis determined by the choice of acquisition function and the dataset. Based on the metrics: Directed Structural Hamming Distance (SHD (D)), Undirected Structural Hamming Distance (SHD (U)), Structural Hamming Distance for the Essential Graphs (PDAG SHD), Non-zero edges predicted (NNZ).

When looking at the scores for the GFlow methods on simulated data, we see that they are similar to the previous section’s. The ECES and EBALD acquisitions functions find a small but non-zero number of edges in their distributions; interestingly, in this case, the EBALD function finds fewer edges than the ECES. Nevertheless, the correct edges found are similar for both algorithms, which leads to the improved SHDs of the EBALD algorithm. Notably, the PDAG SHD score for the EBALD method is the best of all the methods, meaning that the few edges it predicts fit well into the original MEC of the ground truth DAG. In the case of real data, the EBALD method is not stable, and only ECES can find meaningful graphs. However, it also suffers from the problem of predicting too few edges.

### Recommendations for knockout experiments based on the Aging Atlas

To further test our results, we have used GFlow net to learn a DAG distribution based on the Aging Atlas [57]. Based on the distribution, we used each method to predict the top 5 genes worthy for knockout experiments to specify the structure of the GRN.

We have compared these lists with the Aging Atlas and the DAG distribution to find if the predictions are meaningful. We constructed the DAGs based on samples from amphid neurons and used a curated list of 49 genes and the “age” of the organism as variables. The recommended interventions are summarized in Table 5.

**Table 5:** Results on observational data

Method	Interventions				
Uniform	dpy-27	hsp-3	cnx-1	rpl-3	tag-353
Entropy	crh-1	skn-1	hsp-70	ctc-3	pdi-6
BALD	crh-1	skn-1	hsp-70	ctc-3	pdi-6
ECES	crh-1	lin-1	skn-1	hsp-70	F44E5.4
EBALD	crh-1	hsp-70	F44E5.4	F44E5.5	skn-1

Recommened top 5 interventions based on different acquisition funcions.

We can see that crh-1 is consistently recommended across all uncertainty-based acquisition functions. One possible explanation for this, which is mentioned by Roux et al., is that this had the highest correlation of activity and expression, meaning it can act as a confounder for the other variables; thus, knocking this out might give us a clearer picture of the DAG structure. The other common top recommendation is skn-1, which is known to be upregulated in neurons with aging; thus, knocking this out can also show the effects of other important transcription factors, which are less expressed.

## Discussion

We have shown that learning Gene Regulatory Networks with DAG structure learning is feasible and can be achieved in an experimental setting. We used Generative Flow Networks and BayesDAG to learn the structure by conducting simulated experiments. Furthermore, we find that the base predictive performances of the two algorithms are similar, but GFlow can be improved by adding interventional knockout data. We have shown that the choice for the knockout experiments determines the structure of the resulting graph in all cases.

We introduced BALD acquisition for structure learning and compared its performance to Edge Entropy. Notably, the BALD function outperformed Edge Entropy-specifically with the GFlow model-by preserving more high-confidence edges within the final posterior distribution, resulting in more accurate DAG reconstructions. We presented active structure learning in the space of Essential Graphs, or PDAGs. We evaluated the two acquisition functions in this setting as well (ECES and EBALD).

In the case of the BayesDAG, we see that improvement is even less than in the case of the Uniform acquisition function, but the SHD scores for the PDAGs are slightly improved. This suggests that the acquisition strategy may influence edge directionality, thereby improving the PDAG; however, the evidence remains inconclusive and warrants further investigation. In the GFlow models, we obtained more accurate PDAGs, even when accounting for the smaller number of predicted edges.

For the real datasets, the effects are still lagging behind modern structure learning approaches, which are not perfect themselves. However, active learning seems to demonstrate an improvement in the learning process in this case as well. For the validation of the Aging Atlas GRN, we showed that some important genes are found, which can act as latent confounders in the network’s structure, which also demonstrates the power of sequential active data acquisition.

### Complexity and limitations

The limitations of our active learning methods are inherently tied to the structure learning algorithms they are applied to. The additional computational complexity introduced by active learning is minimal. As shown in Eq. 4, after computing the entropy of the distribution, the primary computational overhead consists of an element-wise product of three matrices, which has a complexity of $O(n^2)$. Similarly, entropy computation involves matrix multiplication and a logarithm operation, also achievable in $O(n^2)$. The number of samples per iteration only introduces a constant-factor increase, and the probabilistic selection of interventions scales linearly with a small constant multiplier. Since both structure learning methods already have higher computational complexities, the addition of our active learning framework does not significantly alter their overall runtime.

Our evaluation was conducted on three datasets, two of which had available ground truth graphs. The models exhibited statistically similar performance on both ground truth datasets, suggesting that our performance assessment is reliable within this domain. However, scalability remains a potential limitation. While the method performs well on the tested datasets, its behavior on much larger networks is uncertain, as the increasing number of possible interventions may introduce unforeseen challenges. Furthermore, while the intervention selection strategy based on information-theoretic measures has proven effective in this setting, alternative approaches-such as reinforcement learning—based or hybrid methods—may offer additional gains in efficiency and generalization.

Future work will focus on extending our approach to larger and more complex gene regulatory networks, integrating additional real-world datasets. A possible extension of the work can be toward optimizing scalable algorithms by incorporating biological knowledge [60]. Further analysis will include exploring alternative acquisition functions to improve intervention selection under diverse conditions.

## Conclusion

To summarize, we demonstrate the feasibility of employing active learning strategies to enhance the efficiency and accuracy of Gene Regulatory Network (GRN) structure learning. By leveraging Bayesian approaches, specifically BayesDAG and Generative Flow Networks (GFN), we have shown the potential to improve GRN modeling by integrating interventional data. We evaluated active learning methods, such as BALD and Edge Entropy, and analyzed their effects on structure learning performance. Our results highlight the advantages of the BALD acquisition, particularly in balancing uncertainty reduction and edge prediction accuracy.

In the Essential Graph space, novel acquisition functions such as Equivalence Class Entropy Sampling (ECES) and Equivalence Class Bayesian Active Learning by Disagreement (EBALD) offer promising directions for refining causal discovery methods. While GFN demonstrated significant improvements when interventional data was incorporated, BayesDAG exhibited limited sensitivity to additional data, suggesting areas for future optimization.

This research underscores the importance of tailored acquisition functions in active learning for causal discovery. By focusing on strategies that address the unique challenges of GRN modeling, such as sparsity, high dimensionality, and computational constraints, our approach opens new avenues for theoretical exploration and practical application in systems biology.

Future work could explore hybrid models that combine the strengths of multiple acquisition strategies and multivariate approaches. Furthermore, investigating the scalability of these methods in larger networks and integrating additional biological priors could enhance their applicability.

## Data Availability

The datasets generated and/or analysed during the current study are available in the following repositories: $\bullet$ https://github.com/tschaffter/genenetweaver/tree/master/src $\bullet$ https://www.bnlearn.com/book-crc/code/sachs.discretised.txt.gz $\bullet$ https://www.bnlearn.com/book-crc/code/sachs.interventional.txt.gz $\bullet$ https://c.elegans.aging.atlas.research.calicolabs.com/data

## Code availability

The code is available on the link: https://github.com/sandordani/active_bayes_grn
